# Impact of vascular screening interventions on perceived threat, efficacy beliefs and behavioural intentions: a systematic narrative review

**DOI:** 10.1093/heapro/daad040

**Published:** 2023-06-03

**Authors:** Reindolf Anokye, Ben Jackson, James Dimmock, Joanne M Dickson, Mary A Kennedy, Carl J Schultz, Lauren C Blekkenhorst, Jonathan M Hodgson, Mandy Stanley, Joshua R Lewis

**Affiliations:** Nutrition & Health Innovation Research Institute, School of Medical and Health Sciences, Edith Cowan University, Royal Perth Hospital Research Foundation, Perth, Western Australia, Australia; School of Human Sciences (Exercise and Sport Science), University of Western Australia, Perth, Australia; Telethon Kids Institute, Perth, Western Australia; Department of Psychology, College of Healthcare Sciences, James Cook University, Queensland, Australia; School of Human Sciences (Exercise and Sport Science), University of Western Australia, Perth, Australia; Nutrition & Health Innovation Research Institute, School of Medical and Health Sciences, Edith Cowan University, Royal Perth Hospital Research Foundation, Perth, Western Australia, Australia; School of Arts and Humanities (Psychology Discipline), Edith Cowan University, Joondalup, Western Australia, Australia; Department of Psychological Science, University of Liverpool, Liverpool L69 7ZA, UK; Nutrition & Health Innovation Research Institute, School of Medical and Health Sciences, Edith Cowan University, Royal Perth Hospital Research Foundation, Perth, Western Australia, Australia; Medical School, The University of Western Australia, Perth, Australia; Department of Cardiology, Royal Perth Hospital, Perth,Western Australia, Australia; Nutrition & Health Innovation Research Institute, School of Medical and Health Sciences, Edith Cowan University, Royal Perth Hospital Research Foundation, Perth, Western Australia, Australia; Medical School, The University of Western Australia, Perth, Australia; Nutrition & Health Innovation Research Institute, School of Medical and Health Sciences, Edith Cowan University, Royal Perth Hospital Research Foundation, Perth, Western Australia, Australia; Medical School, The University of Western Australia, Perth, Australia; School of Medical and Health Sciences, Edith Cowan University, Joondalup, Western Australia, Australia; Nutrition & Health Innovation Research Institute, School of Medical and Health Sciences, Edith Cowan University, Royal Perth Hospital Research Foundation, Perth, Western Australia, Australia; Medical School, The University of Western Australia, Perth, Australia; Centre for Kidney Research, Children’s Hospital at Westmead, School of Public Health, Sydney Medical School, The University of Sydney, Sydney, New South Wales, Australia

**Keywords:** cardiovascular imaging, health screening, perceived threat, self-efficacy, response efficacy, behavioural intentions

## Abstract

Health-related behaviours contribute to the global burden of cardiovascular disease (CVD). Cardiovascular imaging can be used to screen asymptomatic individuals for increased risk of CVD to enable earlier interventions to promote health-related behaviours to prevent or reduce CVD risk. Some theories of behaviour and behaviour change assume that engagement in a given behaviour is a function of individual threat appraisals, beliefs regarding the performance of behaviour, self-efficacy for performing the desired behaviour and/or dispositions to act (e.g. behavioural intentions). To date, little is known about the impact of cardiovascular imaging interventions on these constructs. This article summarises evidence related to perceived threat, efficacy beliefs, and behavioural intentions after CVD screening. We identified 10 studies (2 RCTs and 8 non-randomised studies, *n* = 2498) through a combination of screening citations from published systematic reviews and meta-analyses and searching electronic databases. Of these, 7 measured behavioural intentions and perceived susceptibility and 3 measured efficacy beliefs. Findings showed largely encouraging effects of screening interventions on bolstering self-efficacy beliefs and strengthening behavioural intentions. Imaging results that suggest the presence of coronary or carotid artery disease also increased perceived susceptibility to CVD. However, the review also identified some gaps in the literature, such as a lack of guiding theoretical frameworks and assessments of critical determinants of health-related behaviours. By carefully considering the key issues highlighted in this review, we can make significant strides towards reducing CVD risks and improving population health.

## INTRODUCTION

Cardiovascular disease (CVD) is the leading cause of global premature mortality (Nowbar *et al.*, 2019; World Health Organization, 2021). It is estimated by the World Health Organization (WHO) that 80% of premature CVDs can be prevented with modification of lifestyle behaviours such as eating habits, tobacco use, and physical inactivity (WHO and Consultation, 2003; World Health Organization, 2016). Cardiovascular imaging can be used to screen asymptomatic individuals for increased risk of CVD to inform preventive measures (World Health Organization, 2016; World Health Organization, 2017; Kozakova and Palombo, 2020). Several guidelines for primary prevention of CVD (e.g. European guidelines on CVD prevention in clinical practice, US Preventive Services Task Force recommendation statement, American College of Cardiology/American Heart Association guideline for assessment of cardiovascular risk in asymptomatic adults) support the use of cardiovascular imaging for early identification of cardiovascular risk, risk classification and to inform CVD management in asymptomatic adults (US Preventive Services Task Force*, 2009; Greenland *et al.*, 2010; Goff *et al.*, 2014; Piepoli *et al.*, 2016). Individuals identified as having an increased CVD risk due to elevated coronary artery calcium (CAC) scores, carotid plaque or increased carotid intima-media thickness (CIMT) after imaging, are usually advised to make lifestyle modifications including adopting a healthier diet, engaging in regular physical activity, and quitting smoking to potentially reduce the progression of CVD (US Preventive Services Task Force*, 2009; Greenland *et al.*, 2010; Goff *et al.*, 2014; Piepoli *et al.*, 2016; Kozakova and Palombo, 2020).

Some theories of behaviour and behaviour change (e.g. health belief model, protection motivation theory) assume that engagement in a given behaviour is a function of an individual’s judgement regarding the performance of behaviour or value of behavioural performance (e.g. perceived benefits, response efficacy), threat appraisals (e.g. perceived susceptibility and severity), self-efficacy for performing the desired behaviour and/or dispositions to act (e.g. intentions, motivation or willingness to perform a behaviour) (Rosenstock, 1974; Rogers, 1975; Witte, 1992; Fishbein, 2000). Evidence drawn from studies of cancer screening point to the critical role of psychological determinants of behaviours (e.g. self-efficacy or capacity beliefs) in relation to self-management, health-related behaviours and quality of life following screening (Bunge *et al.*, 2008; Aggestrup *et al.*, 2012).

Perceived threat, efficacy beliefs and behavioural intentions are considered key predictors of adaptive health responses and health-related outcomes (Fishbein, 2000; Bunge *et al.*, 2008; Aggestrup *et al.*, 2012). However, little is known about the impact of vascular screening interventions on perceived threat, efficacy beliefs and behavioural intentions. Previous reviews in cardiovascular imaging did not consider the psychological dynamics of behaviour, which has contributed to this knowledge gap (Hackam *et al.*, 2011; Rodondi *et al.*, 2012; Whelton *et al.*, 2012; Mamudu *et al.*, 2014; Gupta *et al.*, 2017). Therefore, it is important to examine the impact of CVD screening interventions on constructs such as threat and efficacy perceptions, and intentions or motivation for behavioural performance. This will provide valuable insights into (i) the determinants of health-related behaviours after screening, (ii) how interventions were received and appraised, and (iii) individual constraints and capabilities to perform a recommended behaviour. These findings will be crucial in promoting healthful behaviours, reducing CVD risks and improving population health. To provide a comprehensive summary of evidence on the impact of cardiovascular screening interventions on perceived threat, efficacy beliefs, and behavioural intentions, we conducted a systematic narrative review.

## METHODS

**Fig. 1:**
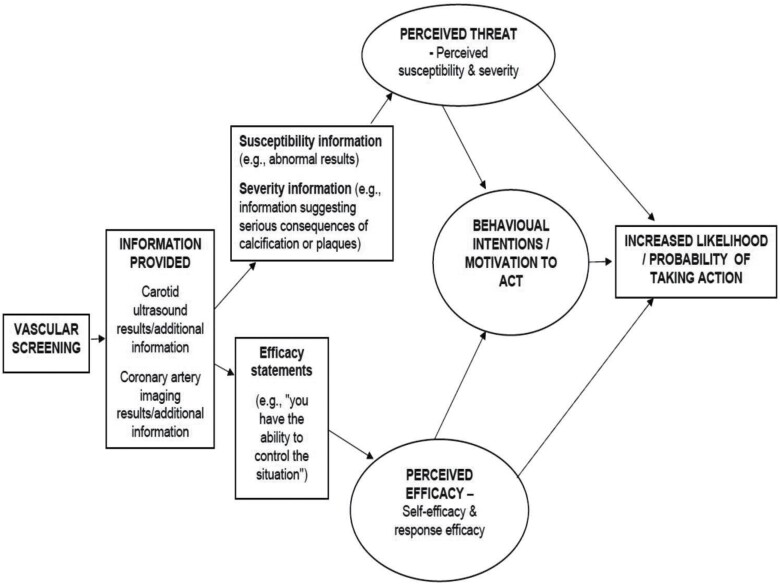
Conceptual model.

A systematic narrative review was considered appropriate to present a scholarly summary of evidence along with interpretation and critique (MacLure, 2005; Grant and Booth, 2009; Greenhalgh *et al.*, 2018). This review was guided by key constructs of the health belief model, protection motivation theory, extended parallel process model, and integrative model of behaviour (Rogers, 1975, 1983; Witte, 1996; Fishbein, 2000; Rosenstock, 2000) (see Figure 1). To inform this review, we conducted a literature search and identified ten relevant studies. The findings are presented using a systematic narrative review approach.

### Search strategy

Relevant articles were retrieved through a combination of screening citations from published systematic reviews and meta-analyses and electronic database searches and manual screening of individual studies retrieved.

#### (a) Citations in systematic reviews and meta-analyses published

We assessed the search strategies and all citations in systematic reviews and meta-analyses published by Hackam *et al.* (Hackam *et al.*, 2011), Rodondi *et al*. (Rodondi *et al.*, 2012), Whelton *et al.* (Whelton *et al.*, 2012), Mamudu *et al.* (Mamudu *et al.*, 2014), and Gupta *et al.* (Gupta *et al.*, 2017). We retrieved and screened articles to identify studies that reported mediators/moderators or psychological determinants of behaviours such as perceived threat, perceived efficacy, and motivation to initiate health-related behaviours.

(b) ***Electronic database searches and manual screening of individual studies retrieved***

An updated systematic search strategy for a previous scoping review (Anokye *et al.*, 2020) was performed (see Appendix 1 in the supplementary file for electronic database searches). We searched MEDLINE, PsychINFO, Social Work Abstracts, Psychology and Behavioural Sciences Collection, and Cumulative Index to Nursing and Allied Health Literature (CINAHL) via EBSCOhost (July 2021) for studies providing non-invasive vascular imaging results to asymptomatic adults to promote healthy lifestyle behaviours. Key search terms included CAC score, carotid ultrasound, carotid plaque, behaviour, lifestyle, motivation, risk perception, medication adherence, and smoking to capture all relevant studies. Manual screening of identified studies was also undertaken to identify eligible studies for this review.

### Inclusion and exclusion criteria

All records were screened for relevance by the first (RA) and last (JRL) authors independently in three stages: (i) Title; (ii) Abstract; and (iii) Full text. Studies were considered relevant if they: (a) were conducted among adults who were at least 18 years old with no pre-existing CVD defined as stroke, myocardial infarction, peripheral arterial disease (PAD), transient ischaemic attack, coronary heart disease, any diagnosed CVD, macrovascular disease, peripheral revascularization; (b) were original, empirical studies conducted among asymptomatic adults (e.g. adults with no physician-diagnosed angina or chest pain and no evidence of intermittent claudication); (c) used non-invasive cardiovascular imaging modality [e.g. carotid ultrasound, computed tomography (CT)] to screen for abnormalities in the arteries and provided screening results to participants (see Supplementary Appendix 2). Included studies also reported at least one outcome of interest (i.e. self-efficacy, response efficacy, perceived susceptibility, perceived severity, motivation to initiate health-related behaviours or plans/intentions/ commitment to engage in health-related behaviours). Health behaviour interventions were considered relevant if they focussed on modifying behavioural risk factors of CVD such as dietary practices, physical activity, smoking and/or alcohol use or improving medication adherence (e.g. lipid-lowering medications including statin, and aspirin) to lower the risk of stroke, heart attack, or other cardiovascular complications.

### Data extraction

Data related to the techniques employed, delivery methods, study setting, population, theories used, target behaviours and follow-up time for outcome assessment were extracted. Data related to outcomes such as perceived susceptibility, perceived self-efficacy, response efficacy and intentions, plans or motivation to modify diet, quit smoking, engage in physical activity, and/or use medication were also extracted. The Grading of Recommendations, Assessment, Development and Evaluations (GRADE) framework was used to evaluate the quality of evidence reported in studies (Guyatt *et al.*, 2011). The studies were rated based on specific limitations, such as incomplete accounting of patients and outcome events, selective outcome reporting bias, use of unvalidated outcome measures, lack of adjustment in statistical analysis, and incomplete follow-up. The studies were then rated as very low, low, moderate, or high based on the GRADE certainty ratings for the risk of bias (Siemieniuk and Guyatt, 2019) (see Appendix 3 in the supplementary file for risk of bias assessment).

## RESULTS

**Fig. 2:**
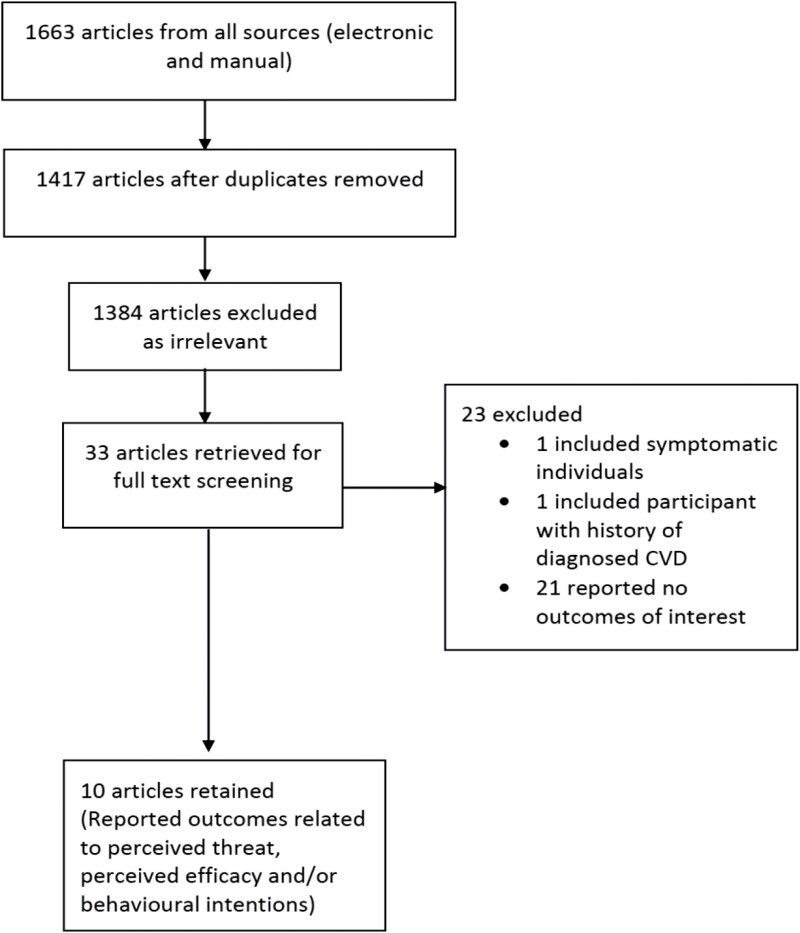
PRISMA flowchart.

Ten studies including asymptomatic adults (*n* = 2498) who were provided with coronary artery screening (2 RCTs, *n* = 888, 4 non-randomised, *n* = 912) or carotid ultrasound results (4 non-randomised, *n* = 698) were included in this review (Figure 2). Of these, seven measured behavioural intentions or motivation to initiate health-related behaviours (i.e. intentions/plans to reduce CVD risk through dietary modifications, smoking cessation, physical activity, and medication usage) (Rupard *et al.*, 2002; Sandwell *et al.*, 2006; Wyman *et al.*, 2007; Korcarz *et al.*, 2008; Rodondi *et al.*, 2008; Johnson *et al.*, 2011; Denissen *et al.*, 2019). Other studies examined the implications of CVD screening on perceived susceptibility (*n* = 7) (Rupard *et al.*, 2002; Sandwell *et al.*, 2006; Wyman *et al.*, 2007; Korcarz *et al.*, 2008; Johnson *et al.*, 2011; Johnson *et al.*, 2015; Schurink *et al.*, 2017), self-efficacy (*n* = 3) (Wyman *et al.*, 2007; Rodondi *et al.*, 2008; Johnson *et al.*, 2011), and response efficacy (*n* = 3) (Wyman *et al.*, 2007; Korcarz *et al.*, 2008; Johnson *et al.*, 2015). None of the studies examined all the five constructs/concepts of interest. One study examined four constructs/concepts (Wyman *et al.*, 2007) and three studies examined three constructs/concepts (Rupard *et al.*, 2002; Korcarz *et al.*, 2008; Johnson *et al.*, 2011). The other studies examined at least one construct/concept. The main target behaviours of the included studies were to increase medication usage/intake or adherence over time, smoking cessation, physical activity, modify dietary habits, and increase physician consultation. Studies were carried out in different settings (community/nonclinical settings, multicentre/different clinical sites, and clinical settings including hospitals, clinics, medical/disease prevention centres, and radiology centres) among smokers, athletes, active-duty army personnel, outpatients, and adults at risk of CVD due to one or more risk factors. Most of the studies were conducted in the USA (*n* = 7) and other studies were conducted in The Netherlands (*n* = 2) and Switzerland (*n* = 1) (see Table 1). Participants included in the selected studies had at least one CVD risk factor (see Table 2). Outcome measures (i.e. perceived threat, efficacy beliefs, behavioural intentions) were assessed immediately and up to 30 months post-screening.

**Table 1:** Overview of included studies

Authors/country	Study design	Setting	Target population	Sample/gender	Imaging Tech.	Follow-up survey	Theory/framework used	Outcomes measured/reported	Target behaviours
Denissen *et al.* Netherlands	Randomised control trial	Community	Adults at risk of CVD due to one or more risk factors	438/*F* = 36%	Multi-detector CT	Average 18 months	None reported	• Desire to reduce CVD risk	Prevention-seeking behaviour (physician consultation) and initiation and compliance with CVD medication
Johnson *et al.* USA	Before-and-after/pre–post design	Multicentre/multiple clinical settings	Outpatients	355/*F* = 58%	Carotid ultrasound	Immediately after scan results	Theory of reasoned action	• Behavioural intentions • Perceived susceptibility • Self-efficacy	Physician decision-making and patient health-related behaviours (dietary, physical activity, smoking, medication intake)
Johnson *et al.* USA	Prospective pre–post study	Radiology centre	Physician-referred and self-referred patients	174/*F* = 38%	Cardiac CT	3 months	Health Belief Model	• Perceived susceptibility • Response efficacy	Health-promoting behaviour change (physical activity, dietary changes, medication usage, smoking cessation)
Korcarz *et al.* USA	Before-and-after/pre-post design	Multicentre/multiple clinical settings	Adults at risk of CVD due to one or more risk factors	263/*F* = 51%	Carotid ultrasound	After scan results	Theory of planned behaviour	• Behavioural intentions • Perceived susceptibility • Response efficacy	Physician treatment plans and patient lifestyle changes (dietary, smoking, physical activity, medication usage)
O’Malley *et al.* USA	Randomised controlled trial	Military base	Active-duty US army personnel	450/*F* = 21%	EBCT	1 year	None reported	Motivation to modify lifestyle	Dietary changes, physical activity, smoking cessation, medication use
Rodondi *et al.* Switzerland	Observational pre-post pilot study	Hospital	Smokers	30/*F* = 43 %	B-mode ultrasound	2 months	None reported	Motivation to cease smoking, self-efficacy	Smoking cessation
Rupard *et al.* USA	Prospective survey study	Medical centre	Smokers	99/*F* = 32%	EBCT	Average 8 months	None reported	• Motivation to cease smoking • Perceived susceptibility • Desire to reduce CVD risk	Smoking cessation
Sandwell *et al.* USA	Prospective cohort study	Community	Community-dwelling adults	364/*F* = 56%	EBCT	6 months	None reported	Perceived susceptibility	Physical activity, dietary changes, medication use
Schurink *et al.* Netherlands	Prospective study	Community	Athletes	275/all men	Non-contrast CT	7–30 months	None reported	Perceived susceptibility	Physician consultation, medication use, lifestyle modification (diet, exercise)
Wyman *et al.* USA	Before-and-after/pre-post pilot design	Multicentre/multiple clinical settings	Outpatients	50/*F* = 38%	Carotid ultrasound	After scan results	Theory of planned behaviour	• Behavioural intentions • Perceived susceptibility • Self-efficacy • Response efficacy	Physician treatment plans and patient lifestyle changes (dietary, smoking, physical activity, medication usage)

**Table 2:** Risk factors measured at baseline for participant inclusion

Authors	Age	DM	Family history of CVD	Smoking	HTN	DLP	HDL-C	Waist Circu.	BMI ≥ 30 kg/m^2^	HbA1c > 6.5%	Minimum criteria for inclusion	% of participants with abnormal results
Denissen *et al.* (2019)	45–74 years		✓	✓				✓^*^	✓		At least 1 CVD risk factor + age	Increased risk (score > 100) = 66% Low risk (score <100)= 34%
Johnson *et al*. (2011)	40–70 years	✓	✓	✓^**^	✓	✓	✓			✓	At least 1 CVD risk factor + age	Carotid plaque—35%
Johnson *et al*. (2015)	*M* = ≥ 45 years *F* = ≥ 55 years	✓	✓	✓^***^	✓		✓				At least 3 CVD risk factors + age	CAC—74%
Korcarz *et al.* (2008)	45–70 years	✓	✓	✓	✓	✓					At least 1 CVD risk factor + age	Carotid plaque–58% CIMT—59%
O'Malley *et al.*, (2003)	39–45 years										Minimum age of 39 years	CAC—15%
Rodondi *et al*. (2008)	40–70 years			✓							Smoking ≥10 cigarettes/day	Carotid plaque—73%
Rupard *et al.* (2002)	Mean 50 years			✓							Active smoker	CAC—42%
Sandwell *et al.* (2006)	≥ 55 years										Minimum age of 55 years	CAC (score <10)—35% CAC(score 11–400)—42% CAC (score >400)—23%
Schurink *et al.* (2017)	≥ 45 years										Minimum age of 45 years	CAC—17%
Wyman *et al.* (2007)	*M* = ≥ 45 years *F* = ≥ 55 years		✓	✓	✓	✓	✓				At least 2 CVD risk factors + age	Carotid plaque—58%

CLM, cholesterol-lowering medications; HTN, hypertension; HCL, hypercholesterolaemia; LDL-C, (low-density lipoprotein) cholesterol; HDL-C, high-density lipoprotein cholesterol; DLP, dyslipidaemia; DM, Diabetes Miletus; HbA1c, Glycosylated Haemoglobin; CIMT, carotid artery intima-media thickness; CAC, coronary artery calcification.

^*^Waist circumference of >88 cm for women/>102 cm for men.

^**^Recent smoking in the past 1 year.

^***^Tobacco use within the past 6 months.

Although all the included studies (*n* = 10) aimed at modifying one or more health-related behaviours, only four studies (Wyman *et al.*, 2007; Korcarz *et al.*, 2008; Johnson *et al.*, 2011; Johnson *et al.*, 2015) reported a theoretical basis (i.e. Health Belief Model, Theory of Planned Behaviour, and Theory of Reasoned Action) (Wyman *et al.*, 2007; Korcarz *et al.*, 2008; Johnson *et al.*, 2011; Johnson *et al.*, 2015). Screening results were provided in different formats including scores (e.g. CAC scores), visuals (e.g. pictures of the arteries, pictures displaying plaque) and a combination of words and graphs (e.g. results in words and a graph format). Others did not describe details of how vascular imaging results were provided and/or the source for further support (see Supplementary Appendix 2). In addition, one intervention (in one study) utilised phone contact as an alternative to in-person individual sessions to provide continuous support (Sandwell *et al.*, 2006). Another intervention (in one study) utilised an educational tutorial (video) to highlight the significance of plaques, the benefits of smoking cessation and CVD risk factors (Rodondi *et al.*, 2008). Most interventions utilised professionals such as dietitians, primary care physicians/general practitioners, or cardiologists, to provide continuous support to change and maintain health-related behaviours (see Supplementary Appendix 2). We provide an overview of evidence regarding the impact of these interventions on perceived threat, perceived efficacy, and behavioural intentions following screening.

## Perceived Threat

Perceived susceptibility and severity (two dimensions of threat) are known to influence preventive behaviours (Brewer *et al.*, 2007; Sheeran *et al.*, 2014). We found seven studies that measured perceived susceptibility following screening in asymptomatic adults (*n* = 1580) (see Table 1) (Rupard *et al.*, 2002; Sandwell *et al.*, 2006; Wyman *et al.*, 2007; Korcarz *et al.*, 2008; Johnson *et al.*, 2011; Johnson *et al.*, 2015; Schurink *et al.*, 2017). Perceived susceptibility to CVD was measured before and after screening in four studies (Wyman *et al.*, 2007; Korcarz *et al.*, 2008; Johnson *et al.*, 2011; Johnson *et al.*, 2015). Outcomes were examined immediately following the provision of screening results in three studies and 3-months after screening in one study (Wyman *et al.*, 2007; Korcarz *et al.*, 2008; Johnson *et al.*, 2011; Johnson *et al.*, 2015). Findings suggest that receiving screening results suggesting calcification or plaques in the coronary or carotid arteries can increase perceived susceptibility to CVD (Wyman *et al.*, 2007; Korcarz *et al.*, 2008; Johnson *et al.*, 2011; Johnson *et al.*, 2015). For example, *Johnson et al*. reported increased perceived susceptibility to CVD among participants in the moderate CVD risk group (CAC scores of 101–400) (*p* = 0.004) (Johnson *et al.*, 2015). Both *Wyman et al.* (Wyman *et al.*, 2007) (mean score pre–post scan, 4.28–3.85 *p* = 0.013) and *Korcarz et al.* (*p* = 0.004) reported increased perceived susceptibility to CVD among participants who were informed about abnormalities in their carotid arteries. *Johnson et al.* (Johnson *et al.*, 2011) also found that participants who received abnormal imaging results were more likely to perceive that they are susceptible to present (OR, 4.14; 95% CI, 1.99–8.62; *p* ≤ 0.001) or future (OR, 2.75; 95% CI, 0.20–1.82; *p* = 0.014) CVD (Johnson *et al.*, 2011).

Three prospective studies also reported increased perceived susceptibility to CVD in participants who were informed about abnormal results (Rupard *et al.*, 2002; Sandwell *et al.*, 2006; Schurink *et al.*, 2017). *Rupard et al*. ( reported that a significantly higher percentage of smokers identified as having abnormal results (17 of 42, 40% vs. 7 of 57, 12%; *p* ≤ 0.01) perceived themselves as having an increased risk of future CVD. *Schurink et al.* found increased perceived susceptibility to CVD in male athletes who received abnormal results (22.9% vs. 4.0%, *p* ≤ 0.001). Moreover, *Sandwellet al*. found higher perceived susceptibility to CVD among participants who were in the moderate and high-risk categories (*p* ≤ 0.001).

It is important to acknowledge that, in most relevant theories, susceptibility perceptions exist alongside appraisals of severity in determining overall threat or fear responses. However, none of the studies included any measure of dimensions of perceived severity (e.g. evaluating perceived medical/clinical consequences such as pain, disability, and death, and/or possible social consequences such as perceived effects on conditions on work, social relations, and family life) following screening.

## Perceived Efficacy

We examine two behavioural theory constructs (i.e. self-efficacy and response efficacy). The construct self-efficacy was measured in three studies that included 435 asymptomatic adults (Wyman *et al.*, 2007; Rodondi *et al.*, 2008; Johnson *et al.*, 2011). Studies refer to self-efficacy as self-perceived confidence, self-confidence, or beliefs concerning the ability to perform a task. We found some interventions and/or studies that showed positive outcomes in terms of improvement in self-efficacy beliefs among participants following screening (Wyman *et al.*, 2007; Rodondi *et al.*, 2008; Johnson *et al.*, 2011). For example, *Wymanet al.* (mean score pre–post scan, 5.85–7.56, *p* = 0.036) and *Johnsonet al.* (*p* = 0.002) reported increased self-efficacy to make lifestyle changes to reduce the risk of stroke and heart disease following screening. Furthermore, *Rodondi et al.* showed increased self-efficacy for smoking cessation after screening from baseline to 2 months in the group with abnormal screening results (mean score pre–post scan, 6.4–7.8, *p* = 0.01).

Response efficacy or outcome expectancies are an important contributor to message acceptance and behaviour tendencies. Three studies measured response efficacy following screening (Wyman *et al.*, 2007; Korcarz *et al.*, 2008; Johnson *et al.*, 2015). We did find one prospective cohort study that showed a positive change in response efficacy after screening (i.e. most participants indicated that making lifestyle changes would reduce their risk for a cardiovascular event) (mean score pre–post scan, 39.7–40.4) (Johnson *et al.*, 2015). In contrast, we observed no noteworthy changes in response efficacy in two studies after screening—using responses to statements such as ‘improving my lifestyle (e.g. eating healthy, exercising regularly) will decrease my heart disease risk’, ‘lowering cholesterol is harmful or beneficial’, and ‘lowering blood pressure is harmful or beneficial’ (Wyman *et al.*, 2007; Korcarz *et al.*, 2008). Findings related to no noteworthy changes in response efficacy or value of behavioural performance raise concerns about existing vascular screening interventions and their potential to elicit health-related behaviours in asymptomatic individuals.

## Behavioural Intentions

We found seven studies that reported outcomes on intentions or plans to engage in exercise and physical activity, initiate dietary changes and/or adhere to medication following screening in asymptomatic adults (*n* = 1599) (Rupard *et al.*, 2002; Sandwell *et al.*, 2006; Wyman *et al.*, 2007; Korcarz *et al.*, 2008; Rodondi *et al.*, 2008; Denissen *et al.*, 2019; Johnson *et al.*, 2011).

### Intentions to engage in physical activity following cardiovascular screening

Intentions to engage in physical activity were reported in three studies (Wyman *et al.*, 2007; Korcarz *et al.*, 2008; Johnson *et al.*, 2011). Results provided slightly conflicting evidence related to the presence of abnormal results in CVD screening and implications for intentions to engage in physical activity. *Johnsonet al.* reported increased intentions to engage in physical activity among participants who received abnormal results (OR, 2.28; 95% CI, 1.24–4.22; *p* = 0.008). *Korcarzet al.* also showed increased intentions to exercise for 30 min, five times each week mostly in the group with abnormal results immediately after screening (absolute difference pre-post scan, 0.714, *p*= < 0.001). However, *Wyman et al.* did not show the same effects of receiving abnormal results on intentions to engage in exercise or reach exercise goals in their study.

### Intentions to initiate dietary changes following cardiovascular screening

Plans or intentions to initiate dietary changes were examined in three studies (Wyman *et al.*, 2007; Korcarz *et al.*, 2008; Johnson *et al.*, 2011). Results provided slightly conflicting evidence related to the presence of abnormal results in CVD screening and implications for intentions to initiate dietary changes.

*Korcarz* *et al.* reported greater increases in plans to lower cholesterol levels by changing diet, greater increases in plans to eat oatmeal, whole-grain bread, cereals, and vegetables (absolute difference pre-post scan, 0.420, *p* ≤ 0.001), and limit foods high in saturated and trans fats such as butter, cheese, ice cream, fatty meats, and deep-fried foods among participants with abnormal results following screening (absolute difference pre–post scan, 0.533, *p* ≤ 0.001). Greater increases in plans to limit intake of sugars, juices, and sweetened drinks, and reduce the quantity of starches in the diet among participants who received abnormal results (absolute difference pre–post scan, 0.272, *p* ≤ 0.001) were also reported. Other outcomes included increased intentions to lower blood pressure by changing diet among participants who received abnormal results (Korcarz *et al.*, 2008). *Johnsonet al.* also reported that abnormal screening findings predicted increased intentions to decrease intake of saturated fat (OR, 2.04; 95% CI, 1.54–2.70; *p* ≤ 0.001), and intentions to modify diet to lower cholesterol levels (OR, 2.95; 95% CI, 1.89–4.61; *p* ≤ 0.001) (Johnson *et al.*, 2011). In contrast, *Wymanet al.* did not observe any significant effect of abnormal results on intentions/plans to reduce blood pressure through dietary changes.

### Intentions to use prescribed medication following cardiovascular screening

Medications such as statins, aspirin, and blood pressure medications are used to control CVD risk factors such as high cholesterol or high blood pressure (Collaboration, 1994; Cannon *et al.*, 2004; LaRosa *et al.*, 2005; Pedersen *et al.*, 2005; Baigent *et al.*, 2009). Cardiovascular screening results appeared to have an effect on strengthening plans or intentions to use prescribed medications to control CVD risk factors (Korcarz *et al.*, 2008; Johnson *et al.*, 2011). *Johnsonet al.* reported that intention to use lipid-modifying medication increased after screening and greater increases were observed in the group that received abnormal results (OR, 19.70; 95% CI, 4.84–80.15; *p* ≤ 0.001). Similarly, *Korcarzet al.* also reported that plans or intentions to use medications to reduce blood pressure and cholesterol significantly increased in participants who received abnormal results (absolute difference pre–post scan, 0.687, *p* ≤ 0.001). *Wymanet al.* also showed trends toward the effect of plaques on intentions or plans to reduce cholesterol through medications. However, no significant effect of screening results on plans to use medications was observed.

### Intentions to cease/quit smoking following cardiovascular screening

Outcomes on intentions, plans, or motivation to cease smoking were reported in five studies (Rupard *et al.*, 2002; Wyman *et al.*, 2007; Korcarz *et al.*, 2008; Rodondi *et al.*, 2008; Johnson *et al.*, 2011). Though most of the studies showed that abnormal results predicted increased intentions to quit/cease smoking, the evidence related to relationships between the type of screening results received, and behavioural intentions were slightly mixed. For example, *Johnsonet al.* reported that abnormal results predicted increased intentions to quit/cease smoking (OR, 4.98; 95% CI, 1.25–19.76; *p* = 0.022). Similarly, *Rodondi et al.* showed increased motivation for smoking cessation, particularly in the group that received abnormal screening results (7.2–8.7, *p* = 0.008). *Rupard et al*. also reported greater increases in motivation to quit smoking among participants with significantly higher CAC scores (mean ± SD = 228 ± 340 vs. 53 ± 76, *p* = 0.028). They also reported that more participants with abnormal results discussed quitting smoking with a physician (45% vs. 42%) and smoked less (plaque present *vs* plaque absent, 57% vs. 48%). However, there were no significant differences in terms of setting quit dates among participants who received abnormal results and those who were informed that they have no abnormal results after screening (Rupard *et al.*, 2002).

### Intentions/disposition to act following cardiovascular screening

Two studies reported outcomes related to participants’ desire to reduce CVD risk following screening (Rupard *et al.*, 2002; Denissen *et al.*, 2019). Studies showed that abnormal results predicted increased desire/commitment to reduce CVD risk. *Denissenet al.* showed that a significant number of participants with increased CAC scores (140/149, 94%) visited a general practitioner following screening as they wished to reduce the risk of CVD and were motivated to undergo a further scan to assess CVD risk. *Rupardet al*. also reported that participants with abnormal results were more likely to discuss imaging results with a personal physician (mean ± SD = 7.4 ± 2.2 vs. 6.3 ± 2.0, *p* = 0.03).

## DISCUSSION

Cardiovascular screening interventions are heterogeneous in the techniques they employ, delivery methods, settings, population, theories used, target behaviours and follow-up time for outcome assessment. We did, however, identify broad limitations in the existing literature that warrant attention in future interventions. Most notably, in behaviour change and fear appeal frameworks overviewed in this article, susceptibility perceptions exist alongside appraisals of severity in determining overall threat or fear responses. However, since no study assessed the perception of severity, it remains unclear whether screening protocols have any noteworthy impact on perceived threat. In addition, some unexpected findings are apparent in the literature. It was noteworthy that screening protocols did not appear (in two studies) to have any effect on participants’ response efficacy perceptions. Also, while abnormal results consistently led to increased perceived susceptibility, differences in behavioural intentions were not always observed among abnormal and normal results groups (Rupard *et al.*, 2002; Wyman *et al.*, 2007). Moreover, we observed relatively little evidence in the literature relating to the application of any theory(ies) to guide the design and delivery of health behaviour change interventions.

Notwithstanding these important considerations, it appears that some studies demonstrated the potential benefits of cardiovascular screening interventions. That is, it appears that these interventions may be responsible for stimulating positive change in key psychological mediators (e.g. perceived susceptibility, self-efficacy, intentions for behaviour change) contributing to healthy lifestyle modification. For example, abnormal results consistently led to increased perceived susceptibility in different populations (e.g. smokers, male athletes, community-dwelling adults, and outpatients). Also, we observed largely encouraging effects of screening interventions on self-efficacy beliefs and behavioural intentions (Rodondi *et al.*, 2008; Johnson *et al.*, 2011; Denissen *et al.*, 2019). It would seem that, at least, in this case, those interventions succeeded in changing self-efficacy beliefs and intentions for behavioural performance.

Evidence suggests that providing risk assessment information to individuals may change perceptions of risk (Miller *et al.*, 2021). Similarly, the findings of this review showed changes in CVD risk perception following screening and provision of imaging results (Wyman *et al.*, 2007; Korcarz *et al.*, 2008; Johnson *et al.*, 2011, Johnson *et al.*, 2015). Findings showed that imaging results suggesting the presence of coronary or carotid artery disease increased CVD risk perception. Perceiving the risk of disease may trigger behavioural modification (Tan *et al.*, 2004). As such, findings underscore the importance of screening for asymptomatic CVD and communication of screening results. A previous study showed that screening can modify health beliefs but may have a limited impact on behavioural intentions (Eiser *et al.*, 2001). In contrast, the findings of this review showed that CVD screening can have a significant impact on behavioural intentions including intentions to cease smoking, engage in physical activity, initiate dietary changes, and use prescribed medication (Rupard *et al.*, 2002; Sandwell *et al.*, 2006; Wyman *et al.*, 2007; Korcarz *et al.*, 2008; Rodondi *et al.*, 2008; Johnson *et al.*, 2011; Denissen *et al.*, 2019).

Psychological determinants of health including self-efficacy beliefs, responses efficacy beliefs, perceived threat and behavioural intentions have been shown to influence health-related behaviours in several studies. Findings from previous studies that examined self-efficacy and protective sexual behaviour, weight loss, exercise and immunizations showed that people rarely attempt behaviour change when self-efficacy is low (Dennis and Goldberg, 1996; Rodgers and Brawley, 1996; AbuSabha and Achterberg, 1997; Cecil and Pinkerton, 1998). Response efficacy plays a significant role in shaping intentions and behaviours and has been shown to influence behavioural outcomes including alcohol consumption and smoking cessation (Floyd *et al.*, 2000; Yun *et al.* 2009). Empirical research also showed a strong link between smoking intentions and smoking behaviours as well as intentions to use medication and medication adherence (Fishbein and Cappella, 2006; Liddelow *et al.*, 2022). However, none of the studies included in this review reported outcomes related to changes in perceived susceptibility, self-efficacy beliefs, response efficacy, behavioural intentions and actual behaviours that could prevent CVD (e.g. smoking, physical activity) after screening. This suggests an important knowledge gap that must be addressed in future CVD screening interventions.

Existing evidence suggests that a significant component of designing, and delivery of health behaviour intervention is the application of psychological and behaviour change theories (Campbell *et al.*, 2000, Campbell *et al.*, 2007; Craig *et al.*, 2008; Glanz and Bishop, 2010). However, we found limited evidence that the design of cardiovascular screening interventions had been informed by relevant theories of behaviour. Interventions are more likely to produce desirable outcomes if they are grounded in appropriate theory (Rimer and Glanz, 2005).

## STRENGTHS AND LIMITATIONS OF THIS REVIEW

We described the current state of research on perceived threat, perceived efficacy, and behavioural intentions or motivation to initiate health-related behaviours in studies using imaging-based screening to improve adherence to behavioural lifestyle recommendations. We summarised evidence regarding key psychological determinants of behaviours and added dimensions of insight that are not available in the existing literature. Key gaps relating to the use of theory and assessment of relevant constructs (i.e. self-efficacy, response efficacy, perceived susceptibility, perceived severity, and behavioural intentions/motivation to initiate health-related behaviours) were also identified. A critical analysis of standing works and strategies (i.e. ‘how-to’ tips) to improve future interventions are also presented.

However, our conclusions regarding the effects of cardiovascular screening interventions on psychological mediators of lifestyle change are based on limited evidence. For example, the potential impact of screening intervention on self-efficacy and response efficacy was rarely examined in the included studies. Also, the construct perceived severity was not examined despite the general appreciation that appraisals of severity exist alongside susceptibility perceptions in determining overall threat or fear responses. None of the studies examined all the five constructs/concepts of interest (i.e. self-efficacy, response efficacy, perceived susceptibility, perceived severity, and behavioural intentions). Furthermore, there’s a possibility that some articles reporting outcomes of interest may have been missed due to database selection and search and/or search strategies used. We focussed on health behaviour interventions that used non-invasive imaging modalities to screen for coronary and carotid artery disease. Moreover, most of the studies were conducted in the USA and therefore findings may differ in other contexts or countries due to differences in culture or attitude which may influence appraisals of threat and efficacy.

## CONCLUSIONS AND FUTURE DIRECTIONS

Findings suggest that screening for asymptomatic CVD and communication of screening findings may be beneficial in terms of helping people form the desired intention to engage in health-related behaviours. In the few instances where perceived susceptibility and self-efficacy beliefs were examined, we observed (largely) encouraging effects of cardiovascular screening interventions on these constructs. For instance, vascular screening results increased perceptions of susceptibility to CVD in different populations. We also identified some gaps in the existing literature that—in seeking to increase the effectiveness of cardiovascular screening interventions—should be addressed in future research. These gaps include assessment to determine how the interventions are received and appraised, and the use of a guiding theoretical framework to inform the scientific process.

We propose that the design of cardiovascular screening interventions is more closely informed by behaviour change theories to, among other things, (i) specify relationships and key constructs to explain the underlying scientific processes of change systematically, (ii) identify and target causal determinants of change and antecedents of behaviour, and (iii) identify mechanisms of action or mediators that may be responsible for intervention effects. We also propose that future studies include assessment (ideally, at multiple time points) of self-efficacy, response efficacy, perceived threat (susceptibility and severity) and intentions to highlight *how* these mechanisms may influence intervention effects. Given both self-efficacy *and* response efficacy are theorised to be important mediators of behaviour change, there is the need for more sensitive measurement in future studies to examine how participants understand the screening process (and results), their ability to perform the recommended action, and/or any mechanisms of action. Early identification of constraints or challenges to behavioural performance can be critical for developing effective strategies to support individuals in performing recommended behaviours. By understanding the barriers that individuals face, strategies can be tailored to address specific challenges and improve the likelihood of success. For individuals who lack motivation or do not intend to perform a behaviour, a strategy might, for instance, involve identifying their values and interests and emphasising how the suggested behaviour aligns with these, or it might involve offering social support and encouragement to help them stay motivated. Moreover, psychological constructs (e.g. self-efficacy) are not fixed traits and can be taught, manipulated and/or enhanced. It is therefore recommended that key components of health behaviour interventions are designed to (i) bolster or increase the strength of beliefs that promote health-related behaviours and/or (ii) reduce the strength of beliefs that promote unhealthy behaviours or eliminate compensatory health beliefs (i.e. beliefs that unhealthy behaviours can be compensated for by subsequent healthy behaviours). Self-efficacy beliefs can be enhanced by providing opportunities for skills building and practice and offering encouragement or support. To improve response efficacy beliefs, individuals must be provided adequate information about the effectiveness of a particular behaviour or action. This can involve sharing evidence-based research, statistics, and data with participants.

## Supplementary Material

daad040_suppl_Supplementary_Appendix_1

daad040_suppl_Supplementary_Appendix_2

daad040_suppl_Supplementary_Appendix_3
