# Supplementary material for: Impact of vascular screening interventions on perceived threat, efficacy beliefs and behavioural intentions: a systematic narrative review
Source: Health Promot Int. 2023 Jun 3;38(3):daad040. doi: 10.1093/heapro/daad040 (PMC10243777; doi:10.1093/heapro/daad040)
Supplement: daad040_suppl_Supplementary_Appendix_1 [file daad040_suppl_supplementary_appendix_1.docx]

| **SEARCH STRATEGY** | | **RESULTS** |
| --- | --- | --- |
| **Database**  MEDLINE  PsychINFO  Social Work Abstracts  Psychology and Behavioural Sciences Collection  Cumulative Index to Nursing and Allied Health Literature (CINAHL)  searched via EBSCOhost | TI ( "coronary stenosis"[MeSH Terms] OR Coronary Stenosis [Text Word] OR Coronary artery stenosis OR "Carotid Stenosis"[MeSH] OR Carotid plaques OR Carotid ultrasound OR Coronary artery calc* OR Coronary calc* OR CAC score* OR Coronary artery calcium score OR Calcium score )  AND  AB ( Mental* OR "Psychological Distress"[MeSH] OR Psych* OR "Quality of Life"[MeSH] OR "Anxiety"[MeSH] OR Anx* OR "Depression"[MeSH] OR Dep* OR mood OR Worr* OR alarm OR Lifestyle change OR Behav* OR Behaviour OR Lifestyle OR Motivation OR Risk perception OR Risk perception* OR Medication adherence OR smoking cessation ) | 1645    Date- 30/07/2021 |

**Appendix 1: Search Strategy**
