# Supplementary material for: Impact of vascular screening interventions on perceived threat, efficacy beliefs and behavioural intentions: a systematic narrative review
Source: Health Promot Int. 2023 Jun 3;38(3):daad040. doi: 10.1093/heapro/daad040 (PMC10243777; doi:10.1093/heapro/daad040)
Supplement: daad040_suppl_Supplementary_Appendix_2 [file daad040_suppl_supplementary_appendix_2.docx]

**Appendix 2: Details of interventions reported in studies included in the review**

| **Authors** | **Intervention components** |
| --- | --- |
| Denissen et al. ^(97)^ | - Imaging results – Provided as CAC score. - Additional information -Explanation of implication of findings. Participants with abnormal results were advised to consult a GP for preventive treatment. - Source/further support – General Practitioner for preventive treatment. |
| Johnson H.M. et al^(89)^ | - Imaging results – Visual format (Pictures of the arteries) - Additional information - Standardised and structured education about the association between abnormal carotid ultrasound results and CVD as well as CVD risk reduction lifestyle recommendations. - Source/further support - Information provided by their primary care physician. |
| Johnson, J.E. et al.^(98)^ | - Imaging results – Provided as CAC score. - Additional information - Explanation of the results and recommendations to follow up with a physician. - Source/further support - Physician |
| Korcarz et al^(91)^ | - Imaging results – Unclear which format results were provided - Additional information – Physicians' recommendations and information suggesting that there's a strong association between carotid artery disease and CVD and that their risk of stroke, heart attack, and death was increased if they had increased CIMT or plaque. - Source/further support – None reported |
| O’Malley et al.^(99)^ | - Imaging results – Visual format (Pictures of CAC findings) - Additional information – Information suggesting that calcification suggests an underlying atherosclerotic coronary artery disease which is predictive of CVD risk. Participants with no coronary artery calcification were cautioned about CVD risk. Dietary, exercise and smoking cessation counselling were provided dependent on assignment to ICM or usual care group. - Source/further support – Referrals to a dietitian, physician, and smoking cessation programme. |
| Rodondi et. al.^(96)^ | - Imaging results – Visual format (Two pictures displaying plaques) - Additional information – Educational tutorial on atherosclerosis highlighting the significance of plaques, the benefits of smoking cessation and CVD risk factors. Participants also received advice for lifestyle modification. - Source/further support – Smoking cessation counselling and Nicotine Replacement Therapy or bupropion at 1 week, 3 weeks, and 2 months |
| Rupard et al.^(92)^ | - Imaging results – Provided as CAC scores - Additional information – Recommendations, individualised counselling for CVD risk factor modification, verbal advice to stop smoking. - Source/further support – Smoking cessation clinic (upon request) |
| Sandwell et al.^(93)^ | - Imaging results – Results provided in words and a graph format - Additional information – A telephone conversation with participants with coronary artery calcification scores >1000 and informed to discuss scan results with their physicians. - Source/further support – Physician consultation |

**Appendix 2 contd.**

| Schurink et al.^(95)^ | - Imaging results – Provided as CAC score - Additional information – Explanation of implication of results and lifestyle recommendations - Source/further support – Some participants (CACS ≥400 AU or coronary stenosis ≥ 50 %) were offered a cardiologist consultation to discuss management options |
| --- | --- |
| Wyman et al^(94)^ | - Imaging results – Visual format (Picture of the artery) - Additional information – Explanation of findings including information that existing evidence suggests a strong association between carotid artery disease and CVD and that the risk of stroke, myocardial infarction and death is higher in individuals with carotid plaque. Counselling related to scan results were also provided. - Source/further support – None reported |
