# Supplementary material for: Impact of vascular screening interventions on perceived threat, efficacy beliefs and behavioural intentions: a systematic narrative review
Source: Health Promot Int. 2023 Jun 3;38(3):daad040. doi: 10.1093/heapro/daad040 (PMC10243777; doi:10.1093/heapro/daad040)
Supplement: daad040_suppl_Supplementary_Appendix_3 [file daad040_suppl_supplementary_appendix_3.docx]

**Appendix 3: GRADE certainty of evidence ratings**

| **Authors (Year)** | **GRADE certainty ratings**  **(Quality of evidence)** |
| --- | --- |
| Denissen et al. (2019) | Moderate |
| Johnson H.M. et al (2011) | High |
| Johnson J.E. et al. (2015) | Moderate |
| Korcarz et al (2008) | High |
| O’Malley et al. (2003) | Moderate |
| Rodondi et al. (2008) | Moderate |
| Rupard et al. (2002) | Moderate |
| Sandwell et al. (2006) | Moderate |
| Schurink et al. (2017) | Moderate |
| Wyman et al (2007) | High |

*High*: there is a high level of confidence that the true effect lies close to the estimate of the effect; *Moderate* : there is moderate confidence in the effect estimate, meaning that the true effect is likely to be close to the estimate, but there is a possibility that it may be substantially different.
